# Diversity of hirudin and hirudin-like factor genes in the North-African medicinal leech, *Hirudo troctina*

**DOI:** 10.1007/s00436-024-08411-x

**Published:** 2024-11-14

**Authors:** Raja Ben Ahmed, Amina Abilov, Christian Müller

**Affiliations:** 1 Department of Biology, Ecology, Biology and Physiology of Aquatic Organisms Laboratory (LR18ES41), Faculty of Sciences of Tunis (FST), University of Tunis El Manar, El Manar Tunis, 2092 Tunis, Tunisia; 2 Animal Physiology, Zoological Institute and Museum, University of Greifswald, 17489 Greifswald, Germany

**Keywords:** Hirudin, Hirudin-like factors, Blood coagulation, Hematophagous leeches

## Abstract

Medicinal leeches of the genus *Hirudo* inhabit large areas of the Palaearctic realm. The distribution range of *Hirudo troctina* includes the southern Iberian peninsula and the northwestern regions of Africa. *H. troctina* is used for medical purposes, but only very little is known about the components of its salivary gland secretion. Hirudins, bivalent inhibitors of thrombin, are probably the best known leech-derived bioactive factors. Hirudin-like factors (HLFs) represent another class of salivary gland components that share characteristic genetic and structural markers with hirudins. Hirudin is not a single entity but exists in at least four different variants. However, there are differences among the European members of the genus *Hirudo* with respect to the actual number of hirudin and HLF genes that are present within their genomes. Here, we describe the identification and molecular cloning of 11 genes that encode for putative hirudin and HLF variants in *H. troctina*. Three of the genes consist of exons and introns that originate from different “archetype” genes and are likely the result of recombination events. The diversity of hirudin and HLF genes in *H. troctina* surpasses that of all other European members of the genus *Hirudo*. The putative hirudin variants and representatives of the HLFs of *H. troctina* were expressed as recombinant proteins, purified and functionally characterized for their thrombin-inhibiting potencies. Phylogenetic analyses based on hirudin and HLF gene sequences of the leech genera *Hirudo*, *Hirudinaria*, and *Whitmania* supported the hypothesis that hirudins and HLFs diverged early in leech evolution.

**Supplementary Information:**

The online version contains supplementary material available at 10.1007/s00436-024-08411-x.

## Introduction

Leeches (Hirudinida) are a taxonomic group of invertebrates belonging to the class Clitellata within the phylum Annelida (Tessler et al. 2018). While some leeches are non-blood-feeding predators, others act as temporary ectoparasites on invertebrates (e.g., snails and crayfish) and various vertebrates including fish, amphibians, reptiles, birds, and mammals (Sawyer 1986). It is likely that hematophagy represents the plesiomorphic state, with independent losses and regainments occurring in various leech lineages over the course of evolution (Siddall et al. 2016; Tessler et al. 2018). Currently, more than 700 leech species have been described (Sawyer 1986; Sket and Trontelj 2008), with only a few classified as “medicinal leeches” (Elliott and Kutschera 2011). These leeches, renowned for their beneficial secretions (Hildebrandt and Lemke 2011), are primarily found within the genus *Hirudo*, which includes the most well-known species used in medicine. In fact, leech anticoagulants, proteins that interfere with normal thrombus formation at various stages of the coagulation cascade, play important roles in modern medicine (Abdualkader et al. 2013; Lemke and Vilcinskas 2020).

Until recently, it was believed that the species *Hirudo medicinalis* described by Linnaeus in 1758 exclusively represented the medicinal leech (for details see Siddall et al. 2007). However, recent investigations have revealed the presence of at least six distinct species within this genus, including *H. medicinalis*, *Hirudo troctina* Johnson, 1816, *Hirudo verbana* Carena, 1820, *Hirudo orientalis* Utevsky & Trontelj 2005, *Hirudo sulukii* Saglam et al. 2016, and *Hirudo nipponia* Whitman, 1886 (Trontelj and Utevsky 2005; Utevsky and Trontelj 2005; Siddall et al. 2007; Saglam et al. 2016). Only very recently, a close relative of *H. nipponia*, namely, *Hirudo tianjinensis* Liu, sp. nov., has been described (Wang et al. 2022). In addition, using an integrative approach that combined morpho-anatomical data with molecular analyses of three genes (COI, 12S rRNA, and ITS2), Arias et al. (2021) discovered two endemic and geographically distinct Iberian lineages of *H. verbana*. One of these lineages was designated as *H. verbana bilineata* ssp. nov., whereas the other lineage was designated as *H. verbana* cf. verbana. It emerged that, despite significant attention in the past and present, our knowledge of medicinal leech diversity remains incomplete.

Furthermore, climate conditions (temperature and precipitation) were suggested to play an important role in the distribution patterns of medicinal leeches (Utevsky et al. 2010; Arias et al. 2021). Within the Mediterranean zone, *H. troctina* is exclusively found in the northwestern regions of Africa and the southern Iberian peninsula (Utevsky et al. 2010; Saglam et al. 2016). A notable feature of this medicinal leech species is its distinctive zigzag-shaped black stripes along the ventro-lateral sides of its body (Hechtel and Sawyer 2002; Ben Ahmed et al. 2015; see Supplementary Information Fig. S1b). In fact, external pigmentation has been argued to be highly useful, perhaps the best characteristic for distinguishing species within the genus *Hirudo* (Hechtel and Sawyer 2002). Additionally, it has been revealed that *Hirudo* species differ from each other in chromosome numbers (Utevsky et al. 2009), saliva composition (Baskova et al. 2008), several molecular markers (Trontelj and Utevsky 2005), and by the diversity of their thrombin inhibitors, especially hirudin variants and hirudin-like factors (HLFs) (Müller et al. 2017).

In fact, the most widely exploited anticoagulant from medicinal leeches is hirudin (Markwardt 1993), a 65 amino acid polypeptide that comprises characteristic features like a molecular mass of about 7 kDa, an isoelectric point (pI) of about 4.2, and the presence of three functionally distinct domains: a short N-terminal sequence of five amino acid residues that blocks the active site of thrombin, a central globular domain that is stabilized by three disulfide bonds, and an elongated C-terminal tail that blocks the fibrinogen-binding site of thrombin (Johnson et al. 1989). HLFs exhibit certain shared features with hirudins, including the presence and arrangement of the six cysteine residues within the central globular domain that are involved in disulfide bond formation, as well as a conserved gene structure that is comprised of four exons and three introns. Nevertheless, hirudins and HLFs may significantly deviate in other aspects, such as molecular masses and isoelectric points (pI values). While certain HLFs display thrombin-inhibitory potencies akin to those of hirudins, others demonstrate either minimal or no discernible thrombin inhibition (Müller et al. 2016, 2020a; Pfordt et al. 2022). To date, four variants of hirudin have been identified in *H. medicinalis*: HV1 (VV) (Dodt et al. 1984), HV2 (IT) (Harvey et al. 1986), and two subvariants of HV3: PAYD (Dodt et al. 1986) and PAFN (Müller et al. 2016). In contrast, *H. orientalis* expresses only the HV 3 subvariants, whereas *H. verbana* expresses both HV1 and the HV3 subvariants but lacks HV2 (Müller et al. 2017).

Several other hirudin-type thrombin inhibitors have been identified in leeches including *H. nipponia* (Hong et al. 1999; Lu et al. 2018; Zhao et al. 2024), *Hirudinaria manillensis* Lesson, 1842 (Steiner et al. 1990; Scacheri et al. 1993; Lukas et al. 2019; Liu et al. 2023), *Macrobdella decora* Say, 1824 (Min et al. 2010; Müller et al. 2019), *Asiaticobdella* (*Aliolimnatis*) *fenestrata* Moore, 1939 (Kvist et al. 2013), *Whitmania pigra* Blanchard, 1887 (Müller et al. 2022; Liu et al. 2024), and *Limnobdella mexicana* Blanchard, 1893 (Iwama et al. 2019; Pfordt et al. 2022).

It was proposed that leeches might exhibit variations in the number of anticoagulants they produce due to their distinct host preferences. It seems that leeches exhibiting broader host specificity tend to comprise a greater diversity of anticoagulant-encoding genes, resulting in a significantly broader and more diverse array of expressed anticoagulants (Kvist et al. 2014, 2016). Notably, although a broad range of anticoagulants have been described in closely related *Hirudo* species, the presence of hirudins and HLFs in *H. troctina* has not yet been confirmed, despite its long-standing use in local traditional and modern medicine.

This study outlines the identification of putative hirudins and HLFs in *H. troctina*. Through successful molecular cloning and subsequent expression, purification and functional characterization of all putative hirudins, and some representatives of HLFs using established coagulation assays, we were able to reveal a surprisingly high diversity of hirudin and HLF genes in *H. troctina* including the presence of several hybrid (or chimeric) genes. Our findings provide new insights into the evolutionary origins of hirudins and HLFs, the molecular processes that contribute to the diversity of leech-derived bioactive factors and support the theory of a singular origin of blood-feeding in leeches.

## Material and methods

### Animal collection, tissue preparation, and genomic DNA isolation

Individuals of *Hirudo troctina* were collected in spring 2023 near the El Malaabi Dam (36.81666 N, 10.98333 E) in Menzel Temime located in the North-East of Tunisia (see Supplementary Information Fig. S1a) and stored in 70% ethanol for long-term preservation. Species identity was confirmed by visual inspection (body coloration pattern, see Supplementary Information Fig. S1b) and molecular genotyping. In detail, partial sequences of the cytochrome c oxidase subunit I gene (COI) were amplified and sequenced using the universal primers LCO1490 and HCO2198 (Folmer et al. 1994; Siddall and Burreson 1998) and compared with databank entries. Genomic DNA of one individual of *H. troctina* was isolated using a small piece of the posterior sucker and the E.Z.N.A. Mollusc DNA Kit (Omega Bio-tek, Inc., Norcross, GA, USA). All steps were carried out as recommended by the manufacturer. Purified genomic DNA was eluted with sterile double distilled water and stored at − 20 °C after determining purity and integrity by agarose gel electrophoresis.

### Cloning of putative hirudin and HLF genes

PCR reactions to amplify the genes of putative hirudins and HLFs were performed using the primers that are listed in Supplementary Information Table S1 and conventional *Taq* polymerase (Thermo Scientific, Schwerte, Germany). Fragments of relevant sizes were purified and cloned into pBluescript KS (Stratagene, San Diego, CA, USA), and their sequences were determined. Sequencing was performed by Biosearch Technologies (LGC, Berlin, Germany). The sequence data of all identified hirudin and HLF genes of *H. troctina* were deposited in GenBank and received the accession numbers PQ050629-PQ050639.

### cDNA synthesis and cloning

cDNA fragments of putative hirudins and HLF variants of *H. troctina* were generated using the gene synthesis service of Synbio Technologies (Monmouth Junction, NJ, USA), amplified using high-fidelity Q5 polymerase (New England Biolabs, Frankfurt a. M., Germany), and cloned into the expression vector pQE30Xa (QIAGEN, Hilden, Germany). Successfully cloned cDNAs were sequenced for control purposes.

### Expression, purification, processing, and quantification of putative hirudins and HLFs

The detailed protocol to express, purify, and quantify the respective putative hirudins and HLFs was described in a couple of recent publications (Müller et al. 2016, 2020a, 2020b; Pfordt et al. 2022). Briefly, we applied an expression and purification system developed by QIAGEN (Hilden, Germany). The pQE30Xa vector encodes a factor Xa protease recognition site located between the His-tag coding region at the 5′ side and the multiple cloning site at the 3′ side. A subsequent Factor Xa protease treatment cleaves off the His-tag and results in a recombinant protein that is devoid of any vector-derived amino acid residues at the N-terminus. Molar concentrations of final protein solutions were calculated by dividing the absorbance at 280 nm by the molar absorption coefficient according to the equation ε = (nW × 5,500) + (nY × 1,490) + (nC × 125) (Gill and von Hippel 1989; Pace et al. 1995).

### Blood coagulation assays

To verify the thrombin-inhibitory potencies of putative hirudins and HLFs, we performed the thrombin time test (TT; reference range 16.8–21.4 s) using a BFT II analyzer (Siemens Healthcare, Erlangen, Germany). All steps were carried out according to the manufacturer’s instructions. Protein samples were diluted with buffer to reach final concentrations in the reaction assays of 3.2 µmol/l, 0.32 µmol/l, or 0.032 µmol/l, respectively. The desired amount of substrate was directly transferred into the test cuvette immediately before the plasma was added. Dade® Ci-Trol® 1 (Siemens Healthcare, Erlangen, Germany) was used as standardized human plasma. The incubation of reaction mixtures was carried out at 37.4 °C. Measurements that exceeded 300 s were stopped and considered as complete inhibition of clot formation.

### Bioinformatics analyses

Basic Local Alignment Search Tool (BLAST) analyses were performed using the respective NCBI web portal and default settings for search algorithm parameters.

Multiple sequence alignments were generated using the CLC Sequence Viewer software package v8.0 (CLC bio, Aarhus, Denmark) and the following settings: gap open cost 5.0, gap extension cost 2.0, and end gap cost: free. Alignments were exported as msf files and further processed using GeneDoc v2.7 (Nicholas and Nicholas 1997). The phylogenetic tree construction was performed using the UPGMA algorithm and the Jukes-Cantor model embedded into the CLC Sequence Viewer software package v8.0. Signal peptide sequences were predicted using the Phobius web server (Käll et al. 2007) and SignalP6.0 (Teufel et al. 2022). The GC content of hirudin and HLF genes was calculated using the GC Content Calculator tool of VectorBuilder (https://en.vectorbuilder.com/tool/gc-content-calculator.html).

## Results

### Identification and characterization of putative hirudin and HLF genes

**Fig. 1:**
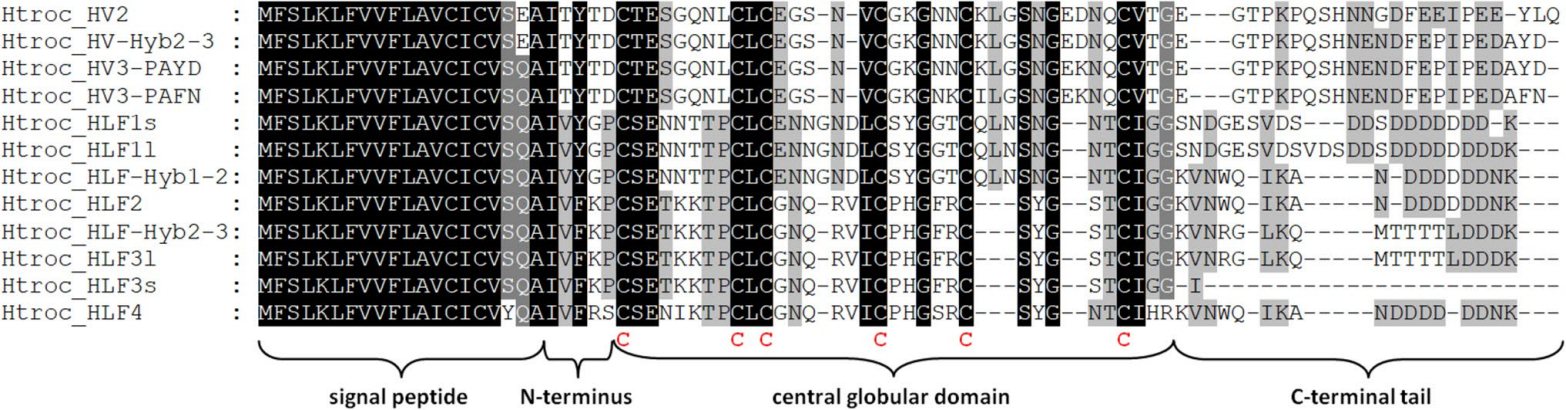
Multiple sequence alignment of hirudin and HLF variants of *H. troctina*. Black background indicates fully conserved amino acid residues; gray background indicates partially conserved amino acid residues. The six conserved cysteine residues giving rise to the three-dimensional structure are marked in bold and red. The signal peptide, the short N-terminus, the central globular domain, and the C-terminal tail are marked with curved brackets. Abbreviations are used according to the IUPAC code

Chromosomal DNA of one genotyped individual of *Hirudo troctina* was subject to PCR reactions using primer pairs that were derived from sequences of hirudin and hirudin-like factor (HLFs) genes of *Hirudo medicinalis*. The respective primer binding sites were located upstream of the putative start codon (forward primers) and downstream of the putative stop codon (reverse primers) to ensure the amplification of the complete exons (coding sequences) and introns. Expected amplicon sizes were about 750 bp (for putative hirudin genes), about 1050 bp (for putative HLF1 genes), and about 900 bp (for putative HLF2-4 genes). Gel analyses revealed the generation of respective PCR products (data not shown). Amplicons of interest were excised, eluted, and cloned into pBluescript KS. Subsequently, recombinant clones were analyzed by restriction analyses, and promising candidates were sent for sequencing. Detailed analyses of the sequencing data led to the identification of four different hirudin and seven different HLF genes in *H. troctina* encoding four putative hirudin variants (namely, HV2, HV3-PAYD, HV3-PAFN, and a hybrid of HV2 and HV3-PAYD) and eight putative HLF variants (namely, HLF1Vlong, HLF1Vshort, HLF2, HLF3long, HLF3short, HLF4, a hybrid of HLF1Vlong and HLF2, and a hybrid of HLF2 and HLF33, respectively). Figure 1 displays a multiple sequence alignment of the deduced amino acid sequences of all putative hirudins and HLFs of *H. troctina*.

All hirudin and HLF genes comprised the typical structure with four exons and three introns (Scacheri et al. 1993; Müller et al. 2016, 2019 and 2022). Exon and intron lengths of all genes as well as the molecular masses and isoelectric points (pI values) of all putative hirudins and HLFs are summarized in Table 1. All values are very close to the respective values of hirudins and HLFs or hirudin and HLF genes of *H. medicinalis* (Müller et al. 2016), *H. verbana*, and *H. orientalis* (Müller et al. 2017).

**Table 1:** Comparison of hirudin and HLF gene structures in terms of exon and intron lengths in bp and GC content values in %. Molecular properties of hirudin and hirudin-like factor variants

Gen	Exon1	Intron1	Exon2	Intron2	Exon3	Intron3	Exon4	GC content	pI	MW
HV2 (IT)	61	103	50	62	76	208	71	33.1	4.12	6.97
HV-Hyb2-3	61	103	50	62	76	225	71	32.9	4.01	6.94
HV3-PAYD	61	103	50	62	76	225	71	32.4	4.31	6.83
HV3 -PAFN	61	103	50	62	76	214	71	32.5	4.35	6.94
HLF1Vshort	61	117	50	71	76	523	62	40.3	3.21	6.44
HLF1Vlong	61	117	50	71	76	522	74	40.2	3.15	6.86
HLF-Hyb1-2	61	117	50	71	76	513	53	38.6	3.89	6.32
HLF2	61	106	50	76	64	383	53	35.8	7.76	6.11
HLF-Hyb2-3	61	106	50	76	64	385	56	35.8	9.08	6.14
HLF3long	61	117	50	76	64	385	56	36.2	9.08	6.14
HLF3short	61	117	50	76	64	364	5	36.0	8.92	4.20
HLF4	61	116	50	71	64	383	53	35.2	7.78	6.27

*pI* isoelectric point, *MW* molecular mass

The sequences of all hirudin and HLF genes of *H. troctina* were deposited in GenBank and received the accession numbers PQ050629-PQ050639.

### Identification of hybrid genes

**Fig. 2:**
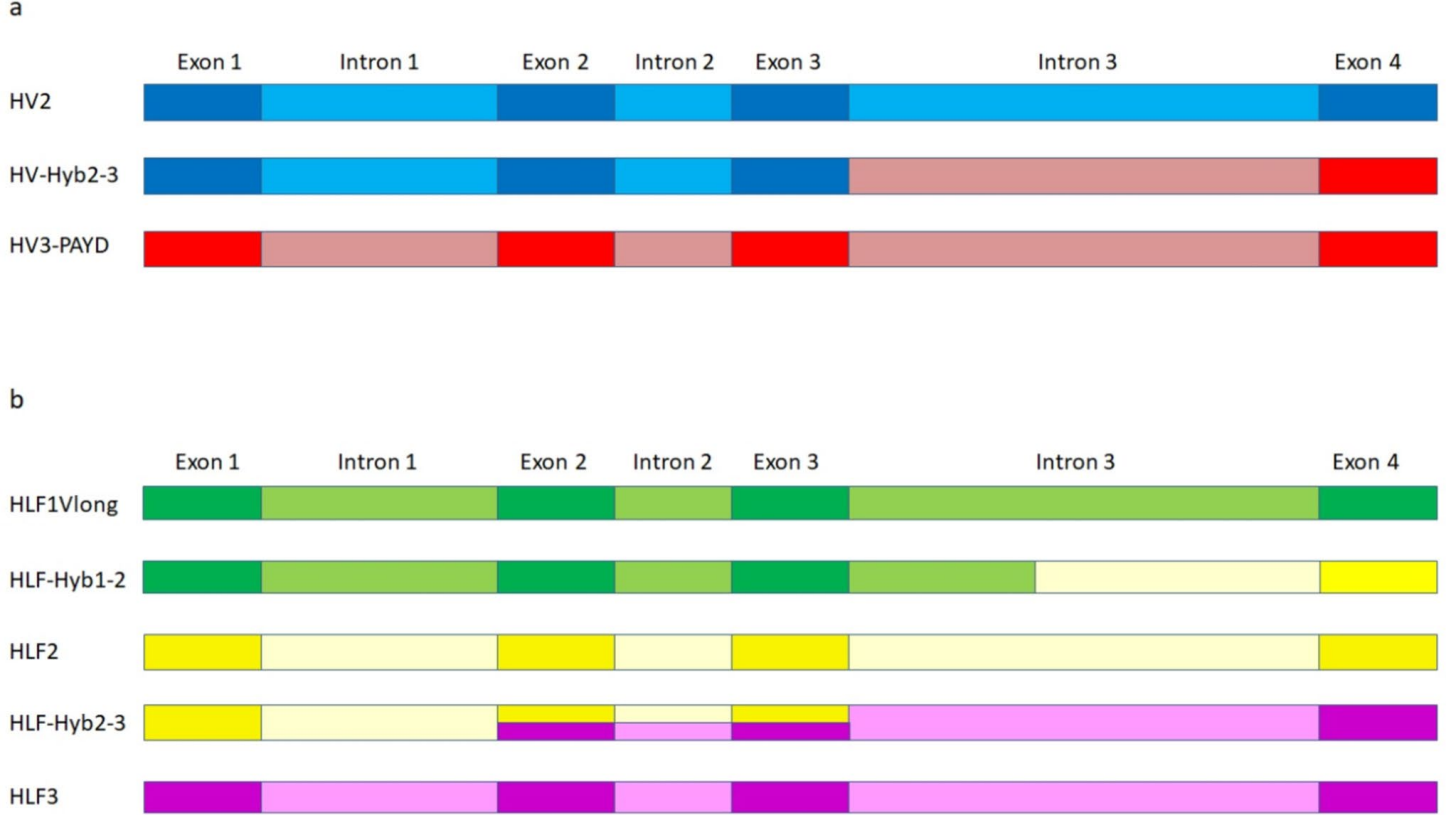
Gene structures of parental and hybrid hirudin and HLF genes of *H. troctina*. Dark colors indicate exons; light colors indicate introns. **a** Schematic representation of the gene structures of HV2 (blue), HV3-PAYD (red), and HV-Hyb2-3 (blue and red; the colors indicate the origin of the respective gene segments). **b** Schematic representation of gene structures of HLF1Vlong (green), HLF2 (yellow), HLF3 (purple), HLF-Hyb1-2 (green and yellow, the colors indicate the origin of the respective domains), and HLF-Hyb2-3 (yellow and purple, the colors indicate the origin of the respective gene segments)

In addition to the “archetype” hirudin and HLF genes, *H. troctina* also comprises several hybrid genes that are composed of exons and introns that originate from different parental genes, namely, HV-Hyb2-3, HLF-Hyb1-2, and HLF-Hyb2-3. Strikingly, all hybrid genes are built up of exons 1–3 and introns 1 + 2 of one parental gene and exon 4 and (partial) intron 3 of the other parental gene (see Fig. 2 and Supplementary Information Fig. S2-S4 for details). Consequently, the deduced amino acid sequences of the respective factors differ in the C-terminal tails that are encoded by exon 4: HV-Hyb2-3 comprises the signal peptide (SP) sequence, the N-terminus, and the central globular domain of HV2 but the C-terminal tail of HV3-PAYD; HLF-Hyb1-2 comprises the SP sequence, the N-terminus, and the central globular domain of HLF1Vlong but the C-terminal tail of HLF2; HLF-Hyb2-3 comprises the SP sequence, the N-terminus, and the central globular domain of HLF2 but the C-terminal tail of HLF3 (see Fig. 1).

### GC content calculation

**Fig. 3:**
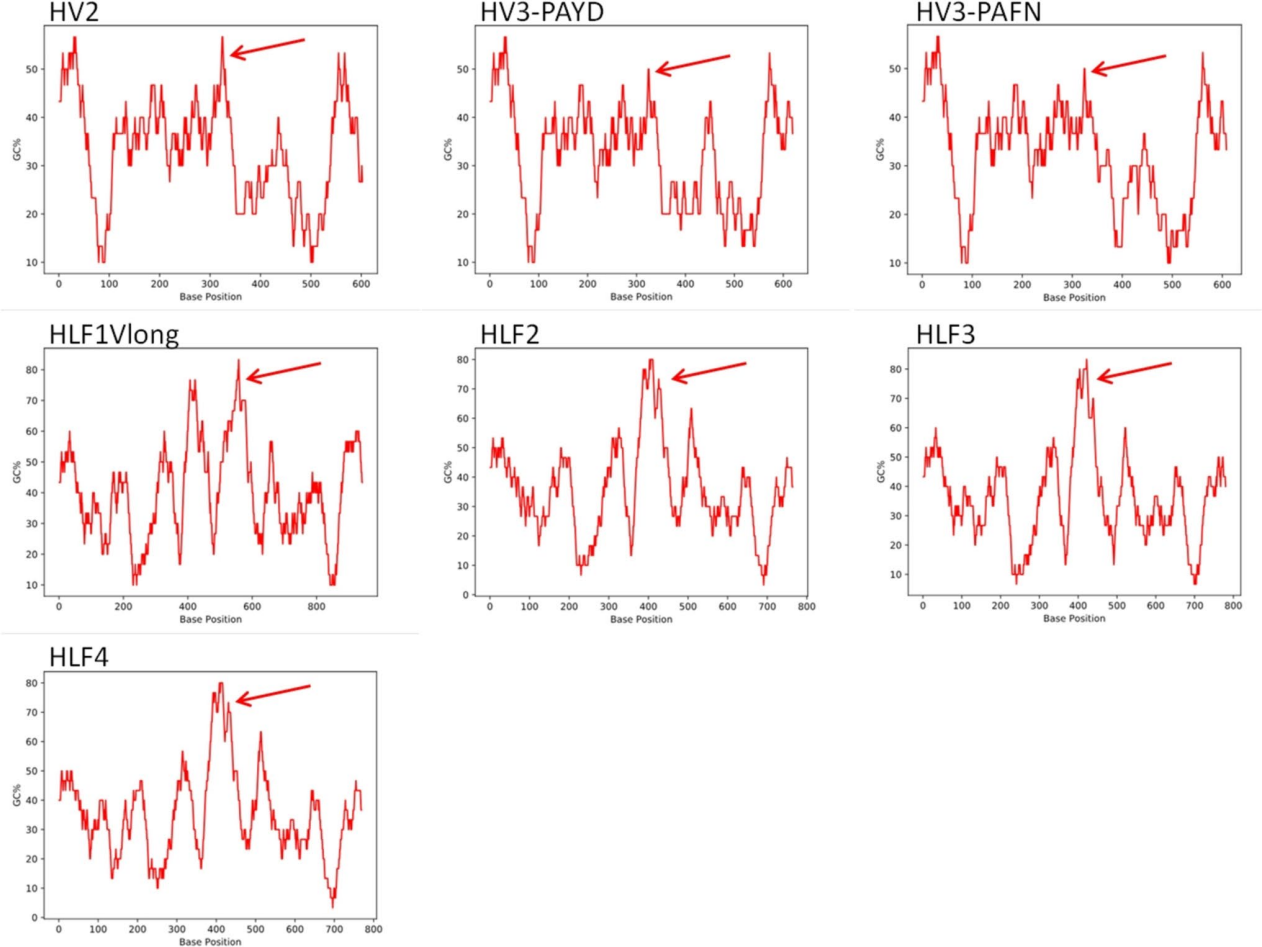
Graphical plots of GC content calculation of hirudin and HLF genes of *H. troctina*. Arrows indicate the exon3/intron3 regions that display the peak in GC values and may represent the local recombination hotspots

The overall GC content values for the genomes of *H. verbana* and *H. medicinalis* are 39% and 36%, respectively (https://www.ncbi.nlm.nih.gov/datasets/genome/GCA_903470615.1/; ~ GCA_011800805.1/; ~ GCA_020137395.1/). An analysis of the hirudin and HLF genes of *H. troctina* revealed GC content values between 32 and 40% (see Table 1). However, the detailed GC plots for all genes indicated the presence of local regions with much higher values that may reach peaks with up to 80% of GC content, especially in the HLF genes (Fig. 3).

### Phylogenetic analyses

**Fig. 4:**
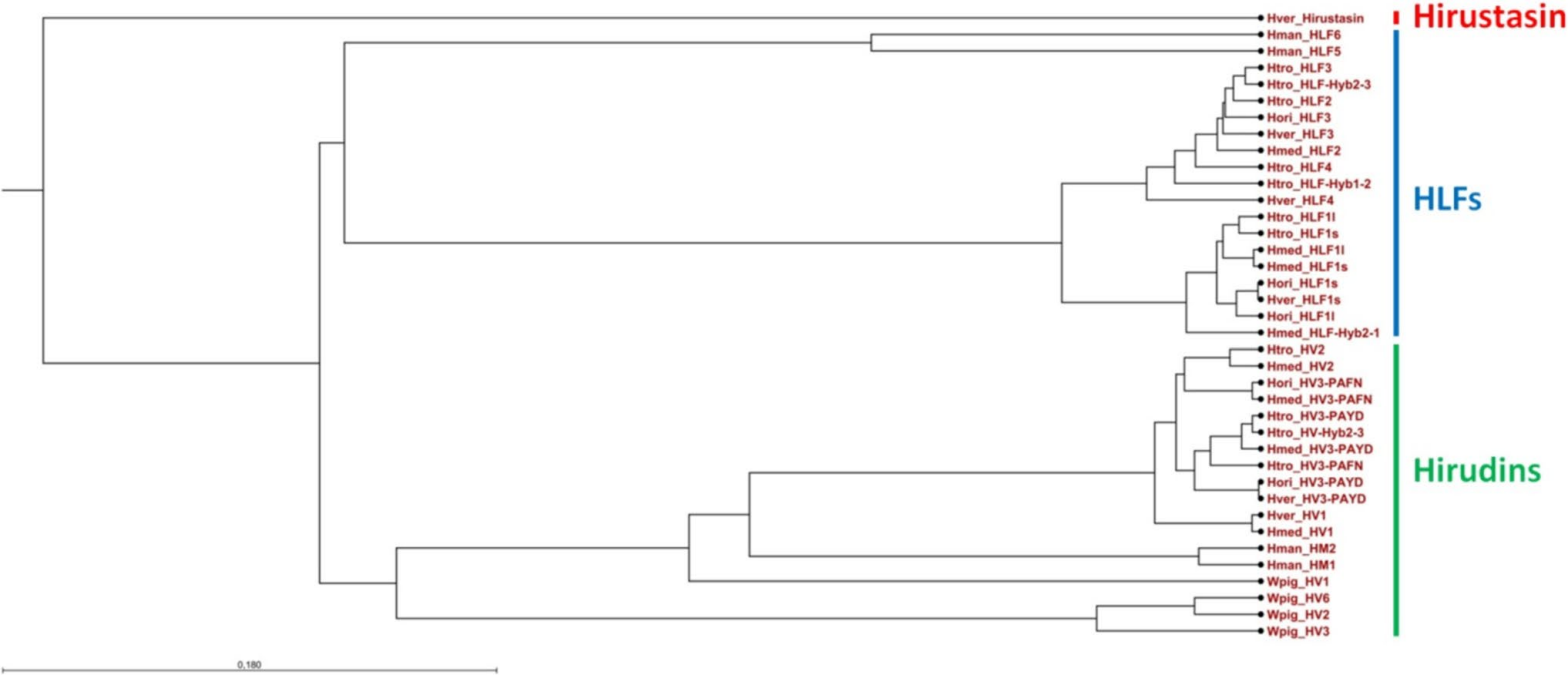
Phylogenetic tree of hirudins, hirudin-like factors (HLFs), and hirustasin based on gene sequences. The scale bar indicates substitutions per site. Hmed, *Hirudo medicinalis*; Hver, *Hirudo verbana*; Hori, *Hirudo orientalis*; Htro, *Hirudo troctina*; Hman, *Hirudinaria manillensis*; Wpig, *Whitmania pigra*

The nucleotide sequences of all hirudin and HLF genes of *H. medicinalis*, *H. verbana,* and *H. orientalis* (Müller et al. 2016 and 2017); the hirudin HM1 + HM2 (Scacheri et al. 1993) and HLF5 + HLF6 genes of *Hirudinaria manillensis* (Müller et al. 2017); and the hirudin genes Wpig_HV1, 2, 3, and 6 of *Whitmania pigra* (Müller et al. 2022) were combined with the hirudin and HLF genes of *H. troctina* for a phylogenetic analysis. The hirustasin gene of *H. medicinalis* (Söllner et al. 1994), a member of the antistasin superfamily (Iwama et al. 2021), was used as the outgroup. The resulting phylogenetic tree revealed a clear separation between hirudin and HLF genes (Fig. 4). Within the hirudins, the HV1 genes were separated from both the HV2 and HV3 genes, and within the HLFs, the HLF1 variant genes were separated from the HLF2-4 genes, respectively. The hirudin genes of *W. pigra* and *H. manillensis* as well as the HLF genes of *H. manillensis* concordantly grouped outside the respective hirudin or HLF genes of the genus *Hirudo*.

### Functional characterization of putative hirudins and HLFs

**Fig. 5:**
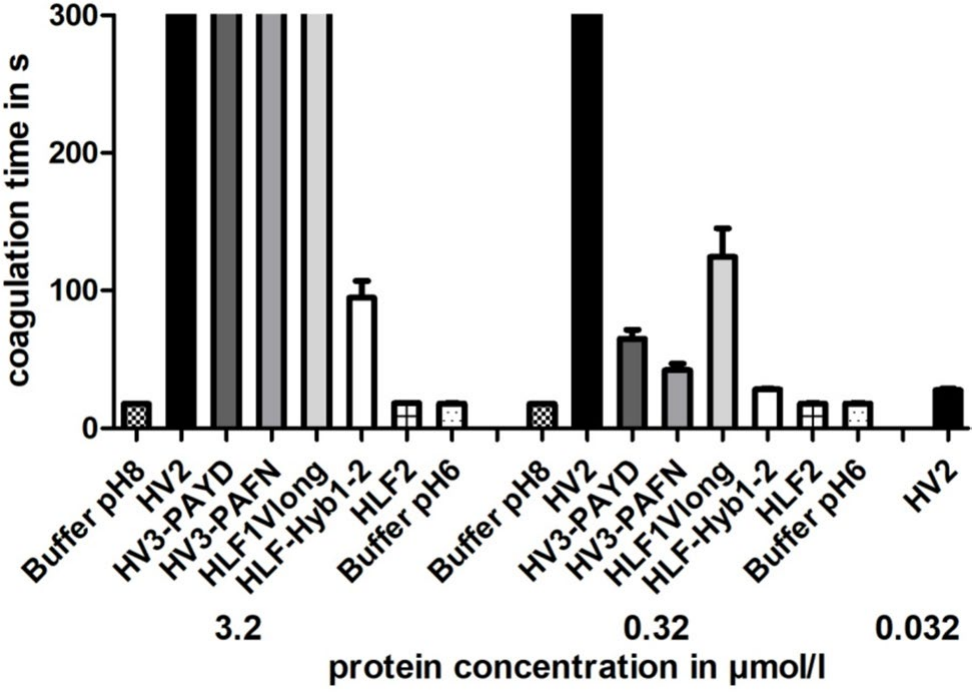
Standard blood coagulation assays of putative hirudin variants HV2, HV2-PAYD, and HV3-PAFN and hirudin-like factor variants HLF1Vlong, HLF-Hyb1-2, and HLF2 of *H. troctina*. Results are means of three independent measurements

To test whether or not the putative hirudins and HLFs of *H. troctina* are thrombin inhibitors, we expressed, purified, and processed the respective factors following an established pipeline as described in the “Material and Methods” section. For practical reasons, we focused on the putative hirudins HV2, HV3-PAYD, and HV3-PAFN as well as the HLFs HLF1Vlong, HLF-Hyb1-2, and HLF2. All hirudins and HLF1Vlong displayed a very high thrombin inhibitory potency, whereas HLF2 did not inhibit the activity of thrombin even at the highest concentration tested (3.2 µmol/l) (see Fig. 5). Interestingly, HLF-Hyb1-2 turned out to be a moderate but clear thrombin inhibitor.

## Discussion

The North-African leech *Hirudo troctina* is a close relative of the European medicinal leech, *Hirudo medicinalis*. Despite its long-lasting use for medical purposes in local traditional and modern medicine, a detailed analysis of its anticoagulant repertoire still remains elusive. In addition, there is not yet a genome project of *H. troctina* to be announced. To get at least a glimpse insight into the spectrum of anticoagulants that is present in *H. troctina*, we applied a targeted molecular approach to identify genes that encode putative hirudins and hirudin-like factors (HLFs). Whereas the sizes of obtained PCR amplicons already indicated that hirudin, HLF1, and HLF2-4 genes are probably present in *H. troctina*, detailed analyses of the sequencing data revealed the presence of not less than 11 different hirudin and HLF genes including four hirudin genes and seven HLF genes, respectively. This is a remarkably high diversity and outnumbers all other European members of the genus *Hirudo* so far (see Table 2 for details).

**Table 2:** Presence ( +) or absence ( −) of genes encoding hirudin and hirudin-like factor variants in members of the genus *Hirudo*

Factor	*H. medicinalis*	*H. verbana*	*H. orientalis*	*H. troctina*
HV1 (VV)	+	+	-	-
HV2 (IT)	+	−	−	+
HV-Hyb2-3	−	−	−	+
HV3-PAYD	+	+	+	+
HV3-PAFN	+	+	+	+
HLF1long	+	+	−	−
HLF1short	+	−	+	−
HLF1Vlong	−	−	+	+
HLF1Vshort	−	+	+	+
HLF1D	−	+	−	−
HLF-Hyb1-2	−	−	−	+
HLF-Hyb2-1	+	−	−	−
HLF2	+	−	−	+
HLF-Hyb2-3	−	−	−	+
HLF3	+	+	+	+
HLF4	−	+	−	+

It has to be mentioned in that context that only one individual of *H. troctina* was investigated, means, all 11 genes were present within the genome of that particular leech. The respective values for *H. medicinalis*, *H. verbana*, and *H. orientalis* are a compilation from different sources including own investigations (Müller et al. 2016, 2017) as well as genome projects *for H. medicinalis* (Babenko et al. 2020; Kvist et al. 2020) and *H. verbana* (Paulsen et al. 2023). It has been proposed that the expression of a broad spectrum of anticoagulants in leeches may be linked to a broad spectrum of putative hosts and vice versa (Kvist et al. 2014, 2016). The feeding preferences of *H. troctina* in that respect are not well investigated, but this might be a very good reason for further investigations.

All hirudin and HLF genes of *H. troctina* comprise the typical “four exon—three intron” structure, and the sizes of all exons and introns are almost identical compared to the respective genes *of H. medicinalis*, *H. verbana*, and *H. orientalis* (see Table 1 and Müller et al. 2016, 2017 for details). A phylogenetic tree that was constructed based on the available gene sequences allocated the hirudins and HLFs of the genus *Hirudo* into different clades. The incorporation of hirudin gene sequences of *W. pigra* (Wpig_HV1, 2, 3, and 6) and hirudin (Himan_HM1 and 2) and HLF (Himan_HLF5 and 6) gene sequences of *H. manillensis* into the analyses retained the overall structure of the resulting tree (see Fig. 4). These results indicate that both hirudin and HLF genes were already present in the last common ancestor of the genera *Hirudo*, *Hirudinaria*, and *Whitmania* and hence support the hypothesis that hirudins and HLFs diverged early in leech evolution (Müller et al. 2017). In addition, our results and data corroborate the assumption that hematophagia represents the ancestral state in leeches (Siddall et al. 2016; Zheng et al. 2023).

The presence of genes, however, does not necessarily indicate that they are indeed expressed. The leech individual that was analyzed in our study was preserved in 70% ethanol. Whereas the quality of the isolated genomic DNA was fairly good, our attempts to isolate intact RNA from the salivary glands failed. It hence still remains to be determined which ones of the many hirudin and HLF genes in *H. troctina* are actually expressed.

Of particular interest in that context are the hybrid genes that were identified in *H. troctina*. HLF-Hyb of *H. medicinalis* (HLF-Hyb2-1 in Table 2 and Fig. 4; a hybrid gene that comprises exons 1–3 of HLF2 and exon 4 of HLF1short; Müller et al. 2016) was the one and only representative of that type of genes identified so far. Our new data indicate that the formation of such type of hybrid genes by local recombination is not an extremely rare event but may represent a more common mechanism to generate genomic diversity in general and diversity of leech hirudin/HLF genes in particular. The great influence of local recombination on human genetic diversity has already been analyzed and discussed by Spencer et al. (2006). Strikingly, in all four cases of leech hybrid genes that were identified so far (HLF-Hyb of *H. medicinalis* and HV-Hyb2-3, HLF-Hyb1-2, and HLF-Hyb2-3 of *H. troctina*, respectively), the putative recombination spots are associated with the respective exon 3/intron 3 region (see Supplementary Information Fig. S2-S4 for details). For the HLF-Hyb1-2 gene, the recombination spot can be narrowed down to a stretch of only 25 base pairs in length that is located within intron 3 (see Supplementary Information Fig. S3 for details). The respective sequence is comparably GC-rich, whereas the overall GC content of all hirudin and HLF genes in *H. troctina* is quite low with values between 32 and 40% (see Table 1 for details). However, in all genes, a peak in GC content is located within intron 3 with values reaching about 60–80% (Fig. 3). It has long been recognized that local rates of recombination in the human genome are positively correlated with an increased GC content (Fullerton et al. 2001). It hence seems possible that the intron 3 of the hirudin and HLF genes in leeches of the genus *Hirudo* may indeed represent an active local recombination hotspot. The exact mode of recombination, however, remains elusive. Generally speaking, recombination may occur during meiosis (Johnston 2024) or mitosis (LaFave and Sekelsky 2009). Mitotic recombination in turn is not restricted to germ line cells but is widespread in somatic cells as well (Siudeja and Bardin 2017). The VDJ rearrangement that generates the diversity of antibodies and T cell receptors in immune cells is probably the most prominent example (Chi et al. 2020). So far, there is no evidence that can be derived from the whole genome data of *H. medicinalis* (Kvist et al. 2020; Babenko et al. 2020) and *H. verbana* (Paulsen et al. 2023) that a similar mechanism is responsible for the diversity of hirudin and HLF genes in European medicinal leeches. Nevertheless, the availability of whole genome data for *H. orientalis*, *H. troctina*, *H. sulukii*, and the non-hematophagous leech *Haemopis sanguisuga* Linnaeus, 1758, would provide a good basis for comprehensive comparative genome analyses and thereby help to better understand the genetic mechanisms behind the diversity of hirudins/HLFs in particular and of anticoagulants in general.

The functional characterization of putative hirudins of *H. troctina* revealed clear evidence that HV2, the HV3 variants, and HLF1Vlong are highly potent thrombin inhibitors, whereas HLF2 displayed no thrombin-inhibitory potency at all, even at the highest concentration tested. These results are in line with previous observations (Müller et al. 2016, 2020a). Of particular interest, however, is the moderate but clearly detectable thrombin-inhibitory potency of HLF-Hyb1-2 (see Fig. 5). The factor comprises the N-terminus and the central globular domain of HLF1Vlong but the C-terminal tail of HLF2 (see Fig. 1 and Supplementary Information Fig. S3). As already pointed out above, the factor HLF-Hyb of *H. medicinalis* has a mirror image-like structure and comprises the N-terminus and the central globular domain of HLF2 but the C-terminal tail of HLF1. Interestingly, HLF-Hyb of *H. medicinalis* has almost no thrombin-inhibitory potency but a synthetic construct (named HLF-Hyb2a) that has exactly the same domain-structure as HLF-Hyb1-2 of *H. troctina* is a potent thrombin inhibitor (Müller et al. 2020a). Our previous and our current observations are hence in very good agreement. HLF3, HLF4, and HLF-Hyb2-3 of *H. troctina* were not purified and functionally characterized but neither HLF3 of *H. medicinalis* nor HLF4 of *H. verbana* displayed any thrombin-inhibitory potency (Müller et al. 2020b). It seems very unlikely that the respective factors of *H. troctina* are thrombin inhibitors. The biological targets of these factors, however, still remain unknown.

## Supplementary Information

Below is the link to the electronic supplementary material.

Supplementary file1 Fig. S1 a) Collection site of H. troctina specimen used in this study. b) Body coloration pattern of H. troctina (left: ventral site; right: dorsal side). Fig. S2 Multiple sequence alignment of H. troctina genes for HV2, HV-Hyb2-3, HV3-PAYD and HV3-PAFNBlack background indicates fully conserved nucleotides; gray background indicates partially nucleotides. The exon sequences are underlined; the GT/AG intron boundaries are marked in italic; the triplets that encode the conserved cysteine residues are marked in yellow; the putative recombination region is marked in green. Abbreviations are used according to the IUPAC code. Fig. S3 Multiple sequence alignment of H. troctina genes for HLF1Vlong, HLF-Hyb1-2 and HLF2. Black background indicates fully conserved nucleotides; gray background indicates partially conserved nucleotides. The exon sequences are underlined; the GT/AG intron boundaries are marked in italic; the triplets that encode the conserved cysteine residues are marked in yellow; the putative recombination region is marked in green. Abbreviations are used according to the IUPAC code Fig. S4 Multiple sequence alignment of H. troctina genes for HLF2, HLF-Hyb2-3 and HLF3. Black background indicates fully conserved nucleotides; gray background indicates partially conserved nucleotides. The exon sequences are underlined; the GT/AG intron boundaries are marked in italic; the triplets that encode the conserved cysteine residues are marked in yellow; the putative recombination region is marked in green. Abbreviations are used according to the IUPAC code. Table S1 List of oligonucleotide primers used in the study (PDF 273 KB)

## Data Availability

Sequence data were deposited at GenBank and are available under the accession numbers PQ050629- PQ050639.
