# Supplementary material for: Diversity of hirudin and hirudin-like factor genes in the North-African medicinal leech, Hirudo troctina
Source: Parasitol Res. 2024 Nov 14;123(11):382. doi: 10.1007/s00436-024-08411-x (PMC11564256; doi:10.1007/s00436-024-08411-x)
Supplement: Supplementary file 1 — Supplementary file1 Fig. S1 a) Collection site of H. troctina specimen used in this study. b) Body coloration pattern of H. troctina (left: ventral site; right: dorsal side). Fig. S2 Multiple sequence alignment of H. troctina genes for HV2, HV-Hyb2-3, HV3-PAYD and HV3-PAFNBlack background indicates fully conserved nucleotides; gray background indicates partially nucleotides. The exon sequences are underlined; the GT/AG intron boundaries are marked in italic; the triplets that encode the conserved cysteine residues are marked in yellow; the putative recombination region is marked in green. Abbreviations are used according to the IUPAC code. Fig. S3 Multiple sequence alignment of H. troctina genes for HLF1Vlong, HLF-Hyb1-2 and HLF2. Black background indicates fully conserved nucleotides; gray background indicates partially conserved nucleotides. The exon sequences are underlined; the GT/AG intron boundaries are marked in italic; the triplets that encode the conserved cysteine residues are marked in yellow; the putative recombination region is marked in green. Abbreviations are used according to the IUPAC code Fig. S4 Multiple sequence alignment of H. troctina genes for HLF2, HLF-Hyb2-3 and HLF3. Black background indicates fully conserved nucleotides; gray background indicates partially conserved nucleotides. The exon sequences are underlined; the GT/AG intron boundaries are marked in italic; the triplets that encode the conserved cysteine residues are marked in yellow; the putative recombination region is marked in green. Abbreviations are used according to the IUPAC code. Table S1 List of oligonucleotide primers used in the study (PDF 273 KB) [file 436_2024_8411_MOESM1_ESM.pdf]

Figure S1

a

b

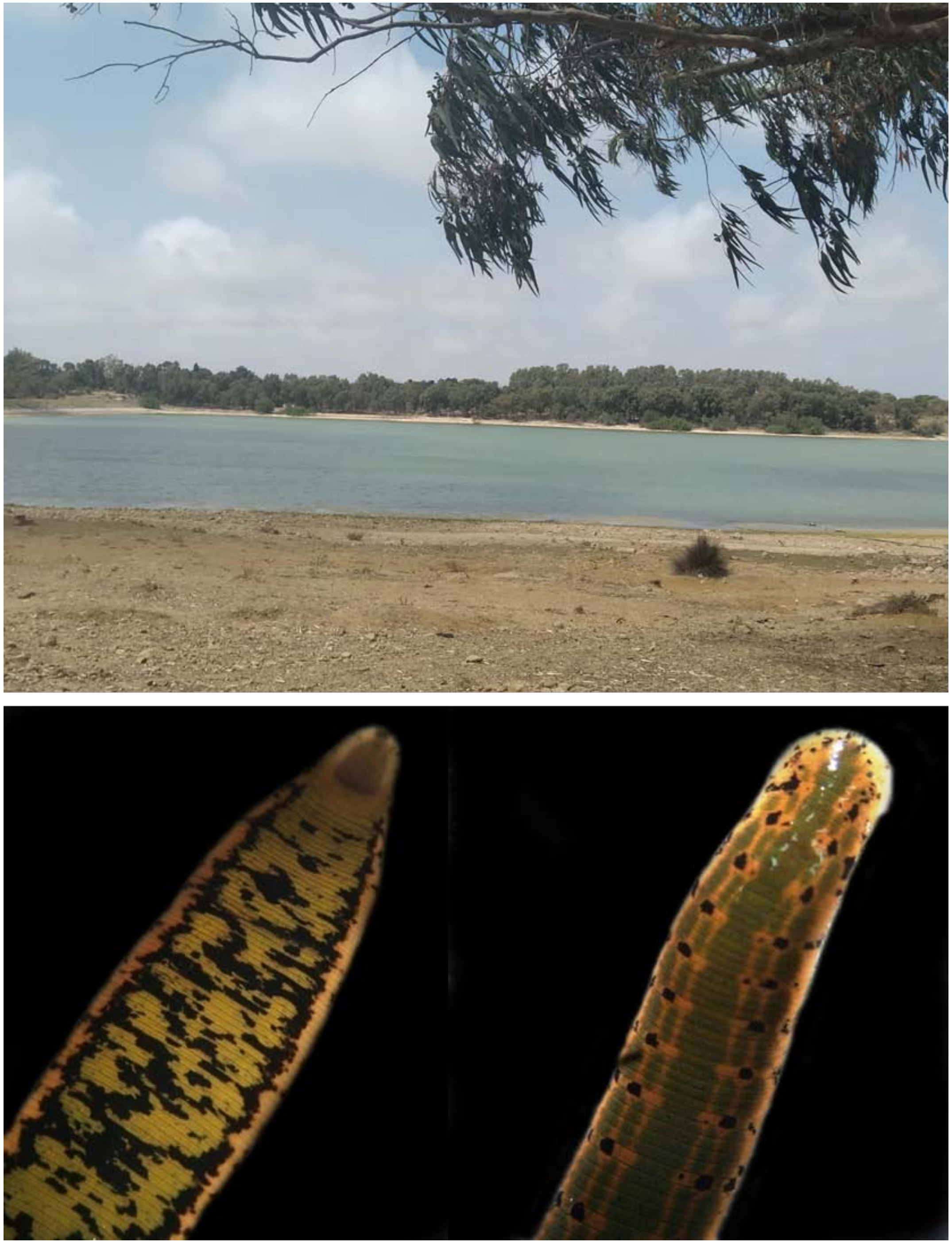

Figure S2

```
                         *        20         *        40         *        60         *        80         *
HV2          : ATGTTCTCTCTGAAGCTGTTCGTTGTCTTCTTGGCGGTTTGCATCTGCGTGTCTGAAGCAAGTGAGTATAAAGTGGACTTTATGAGATTT :  90
HV-Hyb2-3    : ATGTTCTCTCTGAAGCTGTTCGTTGTCTTCTTGGCGGTTTGCATCTGCGTGTCTGAAGCAAGTGAGTATAAAGTGGACTTTATGAGATTT :  90
HV3-PAYD     : ATGTTCTCTCTGAAGCTGTTCGTTGTCTTCTTGGCGGTTTGCATCTGCGTGTCTCAAGCAAGTGAGTATAAAGTGGACTTTATGAGATTT :  90
HV3-PAFN     : ATGTTCTCTCTGAAGCTGTTCGTTGTCTTCTTGGCGGTTTGCATCTGCGTGTCTCAAGCAAGTGAGTATAAAGTGGACTTTATGAGATTT :  90
consensus    : ATGTTCTCTCTGAAGCTGTTCGTTGTCTTCTTGGCGGTTTGCATCTGCGTGTCT AAGCAAGTGAGTATAAAGTGGACTTTATGAGATTT

                     100         *       120         *       140         *       160         *       180
HV2          : AAAATGATTAATTAAAATGTTATGTATATTCCTTCTTTTGTTGGCCGTAGTTGATTAATTGGTTCTCTTTGCAGTTACTTACACTGATTG : 180
HV-Hyb2-3    : AAAATGATTAATTAAAATGTTATGTATATTCCTTCTTTTGTTGGCCGTAGTTGATTAATTGGTTCTCTTTGCAGTTACTTACACTGATTG : 180
HV3-PAYD     : AAAATGATTAATTAAAATGTTATGTATATTCCTTCTTTTGTTGGCCGTAGTTGATTAATTGGTTCTCTTTGCAGTTACTTACACTGATTG : 180
HV3-PAFN     : AAAATGATTAATTAAAATGTTATGTATATTCCTTCTTTTGTTGGCCGTAGTTGATTAATTGGTTCTCTTTGCAGTTACTTACACTGATTG : 180
consensus    : AAAATGATTAATTAAAATGTTATGTATATTCCTTCTTTTGTTGGCCGTAGTTGATTAATTGGTTCTCTTTGCAGTTACTTACACTGATTG

                         *       200         *       220         *       240         *       260         *
HV2          : TACAGAATCGGGTCAAAATTTATGCCTCTGCGAGGTAAGTTAGTAACTTGTGATTCCTACATAACAAATGCTGCAACAAACATTGTTGAA : 270
HV-Hyb2-3    : TACAGAATCGGGTCAAAATTTATGCCTCTGCGAGGTAAGTTAGTAACTTGTGATTCCTACATAACAAATGCTGCAACAAACATTGTTGAA : 270
HV3-PAYD     : TACAGAATCGGGTCAAAATTTATGCCTCTGCGAGGTAAGTTAGTAACTTGTTATTCCTACATAACAAATGCTGCAACAAACATTGTTGAA : 270
HV3-PAFN     : TACAGAATCGGGTCAAAATTTATGCCTCTGCGAGGTAAGTTAGTAACTTGTTATTCCTACATAACAAATGCTGCAACAAACATTGTTGAA : 270
consensus    : TACAGAATCGGGTCAAAATTTATGCCTCTGCGAGGTAAGTTAGTAACTTGT ATTCCTACATAACAAATGCTGCAACAAACATTGTTGAA

                     280         *       300         *       320         *       340         *       360
HV2          : TTGAAGGGAAGCAATGTTTGCGGTAAAGGCAATAATTGCAAATTGGGTTCTAATGGAGAGGACAACCAATGTGTCACTGGCGGTAATTGT : 360
HV-Hyb2-3    : TTGAAGGGAAGCAATGTTTGCGGTAAAGGCAATAATTGCAAATTGGGTTCTAATGGAGAGGACAACCAATGTGTCACTGGCGGTAATTGT : 360
HV3-PAYD     : TTGAAGGGAAGCAATGTTTGCGGTAAAGGCAATAATTGCAAATTGGGTTCTAATGGAGAGAAAAACCAATGTGTCACTGGCGGTAATTGT : 360
HV3-PAFN     : TTGAAGGGAAGCAATGTTTGCGGTAAAGGCAATAAGTGCATATTGGGTTCTAATGGAGAGAAAAACCAATGTGTCACTGGCGGTAATTGT : 360
consensus    : TTGAAGGGAAGCAATGTTTGCGGTAAAGGCAATAAtTGCAaATTGGGTTCTAATGGAGAG A AACCAATGTGTCACTGGCGGTAATTGT

                         *       380         *       400         *       420         *       440         *
HV2          : TTTGACAATTTATAATGTAAACGTTTAAGTTATCACACAGTAT-----TATATAACATTATATCACACCCGAATTTATTAGTTTCTATGA : 445
HV-Hyb2-3    : TTTGACAATTTATAATGTAAACGTTTAAGTTATCACACAGTATTATATAACATTATATTAGACCCGAATTTATTTTATTAGTTTCT--GA : 448
HV3-PAYD     : TTTGACAATTTATAATGTAAACGTTTAAGTTATCACACAGTATTATATAACATTATATTAGACCCGAATTTATTTTATTAGTTTCT--GA : 448
HV3-PAFN     : TTTGACAATTTACAATGCAAACGTCTAAGTTATCACACATTATATTATTATATAACATTATTTCAGACCCGAATTTATTAGTTTCTATGA : 450
consensus    : TTTGACAATTTAtAATGtAAACGTtTAAGTTATCACACAgTAT  tat A AT A ATTA a C gA    A TTTATTAGTTTCT  GA

                     460         *       480         *       500         *       520         *       540
HV2          : AATCACC---TGCCTTATGA---------CCTAATTAATTATATTGGACAAGTTAAAAAAAAG-TGCATAGATATTAATTAATTGTTACT : 522
HV-Hyb2-3    : AATCACTCTTTCCCTCATAAGGGAAATTGCCTAATTAATTATATTAGACAAGTTAAAAAGAAAGTGGATAGATATTAATTAATTGTTACC : 538
HV3-PAYD     : AATCACTCTTTCCCTCATAAGGGAAATTGCCTAATTAATTATATTAGACAAGTTAAAAAGAAAGTGGATAGATATTAATTAATTGTTACC : 538
HV3-PAFN     : AATCACTCTTTCCCTTAAGA---------CCTAATTAATTATATTGGTCTAGTTAAAAAGAAA----ATAGATATTAATTAATTGTTACC : 527
consensus    : AATCACtcttTcCCT At A         CCTAATTAATTATATT GaCaAGTTAAAAAgAAa tg ATAGATATTAATTAATTGTTACc

                         *       560         *       580         *       600         *       620         *
HV2          : AATA-TTTACTAGTTATTAATGTCACATTTTAACTTCAGAAGGTACACCGAAGCCTCAAAGCCATAATAACGGCGATTTCGAAGAAATTC : 611
HV-Hyb2-3    : AATAATTTACTAGTTATTAATATCACATTTTAACTTCAGAAGGTACACCGAAGCCTCAAAGCCATAATGAAAACGATTTCGAACCAATTC : 628
HV3-PAYD     : AATAATTTACTAGTTATTAATATCACATTTTAACTTCAGAAGGTACACCGAAGCCTCAAAGCCATAATGAAAACGATTTCGAACCAATTC : 628
HV3-PAFN     : AATAATTTACTAGTTATTAATATCACATTTTAACTTCAGAAGGTACACCGAAGCCTCAAAGCCATAATGAAAACGATTTCGAACCAATTC : 617
consensus    : AATAaTTTACTAGTTATTAATaTCACATTTTAACTTCAGAAGGTACACCGAAGCCTCAAAGCCATAATgAaaaCGATTTCGAAccAATTC

                     640         *
HV2          : CAGAAGAATATTTACAATGA : 631
HV-Hyb2-3    : CAGAAGACGCTTATGATTAA : 648
HV3-PAYD     : CAGAAGACGCTTATGATTAA : 648
HV3-PAFN     : CAGAAGACGCTTTTAATTAA : 637
consensus    : CAGAAGAcgcTT t AtTaA
```

Figure S3

```
                    *        20         *        40         *        60         *        80         *
HLF1Vlong : ATGTTCTCTCTGAAGCTGTTCGTTGTCTTCTTGGCGGTTTGCATCTGCGTGTCTCAAGCGAGTGAGTATGACCTAACCTTTTTGTGAACG :  90
HLF-Hyb1-2: ATGTTCTCTCTGAAGCTGTTCGTTGTCTTCTTGGCGGTTTGCATCTGCGTGTCTCAAGCGAGTGAGTATGACCTAACCTTTTTGTGAACG :  90
HLF2      : ATGTTCTCTCTGAAGCTGTTCGTTGTCTTCTTGGCGGTTTGCATATGCGTGTCTCAAGCAAGTGAGTATAACCTGACCTTTTTGTGA-CG :  89
consensus : ATGTTCTCTCTGAAGCTGTTCGTTGTCTTCTTGGCGGTTTGCATcTGCGTGTCTCAAGCgAGTGAGTATgACCTaACCTTTTTGTGAaCG

                 100        *       120         *       140         *       160         *       180
HLF1Vlong : AGTATTACGTATTAAAAAGTGCTACATCCTCATAAATACCAGGGTAAATTCACTTCTGCTTTATATTAAAAATGATTGTTGGTTGTAGTT : 180
HLF-Hyb1-2: AGTATTACGTATTAAAAAGTGCTACATCCTCATAAATACCAGGGTAAATTCACTTCTGCTTTATATTAAAAATGATTGTTGGTTGTAGTT : 180
HLF2      : AGTATTACGTAATAAAAAGGGCTA-------ATAAAAG---GGGTAAATTCAATTCTGCTTTATATTAAAAATGGTTGTTGGTTGTAGTT : 169
consensus : AGTATTACGTAtTAAAAAGtGCTAcatcctcATAAAtaccaGGGTAAATTCAcTTCTGCTTTATATTAAAAATGaTTGTTGGTTGTAGTT

                    *       200         *       220         *       240         *       260         *
HLF1Vlong : GTTTATGGACCCTGCTCAGAAAATAATACGACTCCATGCCTGTGTGAGGTAAATTAGAAGATTATATAACATTT-----AATTTTCAAAT : 265
HLF-Hyb1-2: GTTTATGGACCCTGCTCAGAAAATAATACGACTCCATGCCTGTGTGAGGTAAATTAGAAGATTATATAACATTT-----AATTTTCAAAT : 265
HLF2      : GTTTTTAAACCCTGCTCGGAAACTAAAAAGACTCCATGCCTGTGTGGGGTAAATTAGAAGATTATATAACAATTTATTAATTTTCAAAT : 259
consensus : GTTTaTggACCCTGCTCaGAAAaTAAtAcGACTCCATGCCTGTGTGaGGTAAATTAGAAGATTATATAACAtTT     AATTTTCAAAT

                 280        *       300         *       320         *       340         *       360
HLF1Vlong : GTTATCATACATAATATCATCATTAAACAATCAGAATAACGGAAATGATCTATGTTCTTATGGAGGCACGTGCCAGCTGAATTCTAACGG : 355
HLF-Hyb1-2: GTTATCATACATAATATCATCATTAAACAATCAGAATAACGGAAATGATCTATGTTCTTATGGAGGCACGTGCCAGCTGAATTCTAACGG : 355
HLF2      : GTTATCATAAATAATATCATCATTAAACAATCAGAATCA---AAGAGTTATATGCCCTCATGGATTCAGGTGT---------TCTTACGG : 337
consensus : GTTATCATAcATAATATCATCATTAAACAATCAGAATaAcggAAatGaTcTATGttCTtATGGAggCAcGTGccagctgaaTTCTaACGG

                    *       380         *       400         *       420         *       440         *
HLF1Vlong : CAACACATGCATTGGCGGAAGTAATTTTTTTTTACATTGTGTTATTGTACTCAGGACCGCCGAGAAGCAGGGCAGGGGGGCAAGCAGTGG : 445
HLF-Hyb1-2: CAACACATGCATTGGCGGAAGTAATTTTTTTTTACATTGTGTTATTGTACTCAGGACCGCCGAGAAGCAGGGCAGGGGGGCAAGCAGTGG : 445
HLF2      : CAGCACATGCATTGGCGGAAGTAATTTTTTTAT-ATATTGTGTTATTGTATCCAGGACCGCCGAGAAGCAGGGCAGGGGGG---------- : 416
consensus : CAaCACATGCATTGGCGGAAGTAATTTTTTTtTtAcATTGTGTTATTGTActCAGGACCGCCGAGAAGCAGGGCAGGGGGGcaagcagtgg

                 460        *       480         *       500         *       520         *       540
HLF1Vlong : CGCACCCAAACATTTTTCGGGGGAGGGGGGTTTGATTTTTGAATACCATTTTTCTAGGTTTTTTAGGTTTGGGGCGTTAGAACCCCCCAA : 535
HLF-Hyb1-2: CGCACCCAAACATTTTTCGGGGGAGGGGGGTTTGATTTTTGAATACCATTTTTCTAGGTTTTTTAGGTTTGGGGCGTTAGAACCCCCCAA : 535
HLF2      : ------------------------------------------------------------------------------------------ : 416
consensus : cgcacccaaacatttttcgggggaggggggtttgatttttgaataccatttttctaggttttttaggtttggggcgttagaaccccccaa

                    *       560         *       580         *       600         *       620         *
HLF1Vlong : ATTCCCCCTTGGGTGTGCCACTGGGGGAAAGTGCCCCGAGGCCCGGCCCGGCAATTTTTTGGGCCCGCTCGGGTTTTAAAACCTTATTTC : 625
HLF-Hyb1-2: ATTCCCCCTTGGGTGTGCCACTGGGGGAAAGTGCCCCGAGGCCCGGCCCGGCAATTTTTTGGGCCCGCTCGGGTTTTAAAACCTTTTTTC : 625
HLF2      : -----------------------AGGGCAAGTGCCCCG------GGACCGGCAATTTTTGGGGCCCGCTCGGGTTTTAAAACCTTTTTTC : 477
consensus : attcccccttgggtgtgccactggGGGaAAGTGCCCCGaggcccGGcCCGGCAATTTTTtGGGCCCGCTCGGGTTTTAAAACCTTtTTTC

                 640        *       660         *       680         *       700         *       720
HLF1Vlong : ATCTCCAATACCAAATTGATAAATTGATATTACCAAGGGGCCCGAAATTTGTTTCGGCGGATGTGATTGTACTTATGTACTTGTGATTGT : 715
HLF-Hyb1-2: TTCTCCAATACCAAATTGATAAATTGATAT-----AGGGGCCCGAAAGTTGTTTCGGCGGACCTGATTGTGTCTGTATT------ATTGA : 704
HLF2      : TTCTCCAATACCAAATTGATAAATTGATAT-----AGGGGCCCGAAAGTTGTTTCGGCGGACCTGATTGTGTCTGTATT------ATTGA : 556
consensus : tTCTCCAATACCAAATTGATAAATTGATAT     AGGGGCCCGAAAgTTGTTTCGGCGGAccTGATTGTgtcTgTaTt      ATTGa

                    *       740         *       760         *       780         *       800         *
HLF1Vlong : TACTATTATGCTCTTAACCTGTTATTAATTGTTAGAAGCACATCGTATCTAAAGCTGAAGTTTTATGAATTGCCATTTGCTGCTGAGAGT : 805
HLF-Hyb1-2: TACCGTTATGCTCTTAACTTGTTATTAATTATTAGAGGCACATTGTGTCTAAAACTGAAATTTATTGGATTGCTATTTATTGCTCAGATT : 794
HLF2      : TACCGTTATGCTCTTAACTTGTTATTAATTATTAGAGGCACATTGTGTCTAAAACTGAAATTTATTGGATTGCTATTTATTGCTCAGATT : 646
consensus : TACcgTTATGCTCTTAACtTGTTATTAATTaTTAGAgGCACATtGTgTCTAAAaCTGAAaTTTatTGgATTGCtATTTatTGCTcAGAtT

                 820        *       840         *       860         *       880         *       900
HLF1Vlong : TAACAATCTCCAACACAAG--TGCTGACAAATCCTTTGATTTTGTAATGAAATATCAATTTTTAAATATTAATCCTTAAATTTCGTTTCG : 893
HLF-Hyb1-2: TATTAATCTGCAGCACACGGTTGCTAAAAATCTTTTTAGTTTTGTAATGAAATAATATATTTTTAAATTTAAATTCTGAAATGTCGTTTTG : 884
HLF2      : TATTAATCTGCAGCACACGGTTGCTAAAAATCTTTTTAGTTTTGTAATGAAATAATATATTTTTAAATTTAAATTCTGAAATGTCGTTTTG : 736
consensus : TAttAATCTgCAgCACAcGgtTGCTaAaAAtctttTTagTTTTGTAATGAAATAatAtTTTTTAAATtTaAATtCTgAAATgTCGTTTtG

                    *       920         *       940         *       960         *
HLF1Vlong : ACAGGCAATGACGGTGAGTCAGTCGACAGTGTCGACAGTGACGACAGTGACGACGACGACGACGATGATGATAAATAA : 971
HLF-Hyb1-2: ACAGA-----------AGTTAACTGGCA--------AATTAAAGCAA--ATGACGACGACGACGACGATAATAAATAA : 941
HLF2      : ACAGA-----------AGTTAACTGGCA--------AATTAAAGCAA--ATGACGACGACGACGACGATAATAAATAA : 793
consensus : ACAGa           AGTtAactGgCA        AaTtAaagCAa  AtGACGACGACGACGAcGATaATAAATAA
```

Figure S4

```
                    *        20         *        40         *        60         *        80         *
HLF2       : ATGTTCTCTCTGAAGCTGTTCGTTGTCTTCTTGGCGGTTTGCATATGCGTGTCTCAAGCAAGTGAGTATAACCTGACCTTTTTGTGA-CG :  89
HLF-Hyb2-3: ATGTTCTCTCTGAAGCTGTTCGTTGTCTTCTTGGCGGTTTGCATATGCGTGTCTCAAGCAAGTGAGTATAACCTGACCTTTTTGTGA-CG :  89
HLF3       : ATGTTCTCTCTGAAGCTGTTCGTTGTCTTCTTGGCGGTTTGCATCTGCGTGTCTCAAGCGAGTGAGTATGACCTAACCTTTTTGTGAACG :  90
consensus : ATGTTCTCTCTGAAGCTGTTCGTTGTCTTCTTGGCGGTTTGCATaTGCGTGTCTCAAGCaAGTGAGTATaACCTgACCTTTTTGTGA CG

                   100         *       120         *       140         *       160         *       180
HLF2       : AGTATTACGTAATAAAAAGGGCTA-------ATAAA---AGGGGTAAATTCAATTCTGCTTTATATTAAAAATGGTTGTTGGTTGTAGTT : 169
HLF-Hyb2-3: AGTATTACGTAATAAAAAGGGCTA-------ATAAA---AGGGGTAAATTCAATTCTGCTTTATATTAAAAATGGTTGTTGGTTGTAGTT : 169
HLF3       : AGTATTACGTATTAAAAAGTGCTACATCCTCATAAATACCAGGGTAAATTCACTTCTGCTTTATATTAAAAATGGTTGTTGGTTGTAGTT : 180
consensus : AGTATTACGTAaTAAAAAGgGCTA       ATAAA   agGGGTAAATTCAaTTCTGCTTTATATTAAAAATGGTTGTTGGTTGTAGTT

                    *       200         *       220         *       240         *       260         *
HLF2       : GTTTTTAAACCCTGCTCGGAAACTAAAAAGACTCCATGCCTGTGTGGGGTAAATTAGAAGATTATATAACAATTTTATTAATTTTCAAAT : 259
HLF-Hyb2-3: GTTTTTAAACCCTGCTCGGAAACTAAAAAGACTCCATGCCTGTGTGGGGTAAATTAGAAGATTATATAACAATTTTATTAATTTTCAAAT : 259
HLF3       : GTTTTTAAACCCTGCTCGGAAACTAAAAAGACTCCATGCCTGTGTGGGGTAAATTAGAAGATTATATAACAATTTTATTAATTTTCAAAT : 270
consensus : GTTTTTAAACCCTGCTCGGAAACTAAAAAGACTCCATGCCTGTGTGGGGTAAATTAGAAGATTATATAACAATTTTATTAATTTTCAAAT

                   280         *       300         *       320         *       340         *       360
HLF2       : GTTATCATAAATAATATCATCATTAAACAATCAGAATCAAAGAGTTATATGCCCTCATGGATTCAGGTGTTCTTACGGCAGCACATGCAT : 349
HLF-Hyb2-3: GTTATCATAAATAATATCATCATTAAACAATCAGAATCAAAGAGTTATATGCCCTCATGGATTCAGGTGTTCTTACGGCAGCACATGCAT : 349
HLF3       : GTTATCATAAATAATATCATCATTAAACAATCAGAATCAAAGAGTTATATGCCCTCATGGATTCAGGTGTTCTTACGGCAGCACATGCAT : 360
consensus : GTTATCATAAATAATATCATCATTAAACAATCAGAATCAAAGAGTTATATGCCCTCATGGATTCAGGTGTTCTTACGGCAGCACATGCAT

                    *       380         *       400         *       420         *       440         *
HLF2       : TGGCGGAAGTAATTTTTTATATATTGTGTTATTGTATCCAGGACCGCCGAGAAGCAGGGCAGGGGGGAGGG-CAAGTGCCCCGGGACCGG : 438
HLF-Hyb2-3: TGGCGGAAGTAATTTTTTATATATTGTGTTATTGTATCCAGGACCGCCGAGAAGCAGGGCAGGGGGGAGGGGCAAGTGCCCCGGGACCGG : 439
HLF3       : TGGCGGAAGTAATTTTTTATATATTGTGTTATTGTATCCAGGACCGCCGAGAAGCAGGGCAGGGGGGAGGGGCAAGTGCCCCGGGACCGG : 450
consensus : TGGCGGAAGTAATTTTTTATATATTGTGTTATTGTATCCAGGACCGCCGAGAAGCAGGGCAGGGGGGAGGGgCAAGTGCCCCGGGACCGG

                   460         *       480         *       500         *       520         *       540
HLF2       : CAATTTTTGGGGCCCGCTCGGGTTTTAAAACCTTTTTTCTTCTCCAATACCAAATTGATAAATTGATATA-----GGGGCCCGAAAGTTG : 523
HLF-Hyb2-3: CAATTTTTGGGGC----TCGGGTTTTAAAACCTTTTTTCTTCTCCAATACCAAATTGATAAATTGATATAATATAGGGGCCCGAAAGTTG : 525
HLF3       : CAATTTTTGGGGC----TCGGGTTTTAAAACCTTTTTTCTTCTCCAATACCAAATTGATAAATTGATATAATATAGGGGCCCGAAAGTTG : 536
consensus : CAATTTTTGGGGC    TCGGGTTTTAAAACCTTTTTTCTTCTCCAATACCAAATTGATAAATTGATATAatataGGGGCCCGAAAGTTG

                    *       560         *       580         *       600         *       620         *
HLF2       : TTTCGGCGGACCTGATTGTGTCTGTATTATTGATACCGTTATGCTCTTAACTTGTTATTAATTATTAGAGGCACATTGTGTCTAAAACTG : 613
HLF-Hyb2-3: TTTCGACGGACCTGATTGTGTCTGTATTATTGATACTGTTATGCTCTTAACTTGTTATTAATTATTAGAGGCACATTGTGTCTAAAGCTG : 615
HLF3       : TTTCGACGGACCTGATTGTGTCTGTATTATTGATACTGTTATGCTCTTAACTTGTTATTAATTATTAGAGGCACATTGTGTCTAAAGCTG : 626
consensus : TTTCGaCGGACCTGATTGTGTCTGTATTATTGATACtGTTATGCTCTTAACTTGTTATTAATTATTAGAGGCACATTGTGTCTAAAgCTG

                   640         *       660         *       680         *       700         *       720
HLF2       : AAATTTATTGGATTGCTATTTATTGCTCAGATTTATTAATCTGCAGCACACGGTTGCTAAAAATCTTTTTAGTTTTGTAATGAAATAATA : 703
HLF-Hyb2-3: AAATTTATTGGATTGCTATTTATTGCTCAGATTTAACAATCTGCAGCACACGCTTGCTAAAAATCTTTTTAGTTTTGTAATGAAATAATA : 705
HLF3       : AAATTTATTGGATTGCTATTTATTGCTCAGATTTAACAATCTGCAGCACACGCTTGCTAAAAATCTTTTTAGTTTTGTAATGAAATAATA : 716
consensus : AAATTTATTGGATTGCTATTTATTGCTCAGATTTAacAATCTGCAGCACACGcTTGCTAAAAATCTTTTTAGTTTTGTAATGAAATAATA

                    *       740         *       760         *       780         *       800         *
HLF2       : TTTTTTAAATTTAAATTCTGAAATGTCGTTTTGACAGAAGTTAACTGGCAAATTAAAGCAAATGACGACGACGAC----GACGATAATAA : 789
HLF-Hyb2-3: TTTTTTAAATTTAAAGTCTGAAATGTCGTTTCGACAGAAGTTAACAGG-GGATTAAAGCAAATGACGACGACGACACTAGACGATGATAA : 794
HLF3       : TTTTTTAAATTTAAAGTCTGAAATGTCGTTTCGACAGAAGTTAACAGG-GGATTAAAGCAAATGACGACGACGACACTAGACGATGATAA : 805
consensus : TTTTTTAAATTTAAAgTCTGAAATGTCGTTTcGACAGAAGTTAACaGG ggATTAAAGCAAATGACGACGACGACactaGACGATgATAA

HLF2       : ATAA : 793
HLF-Hyb2-3: ATAA : 798
HLF3       : ATAA : 809
consensus : ATAA
```

Table S1

List of Primers

Primers used for genotyping (Folmer et al., 1994):

LCO1490:	5'-GGT CAA CAA ATC ATA AAG ATA TTG G-3'
HC02198:	5'-TAA ACT TCA GGG TGA CCA AAA AAT CA-3'

Primers used for gene amplification:

hirudin genes:	forward:	5′-GGC ACG AGG GAT CTG AAG AAATC-3′
	reverse:	5′-TAT TGG TAA ATA GCT TAG CTATGG-3′
HLF genes:	forward:	5′-GGC AGG TAC ATC ATT CTG ATC TG-3′
	reverse:	5′-GCT TTATGA CGT CAT AAC TTTATT GG-3′

Primers used for cloning in pQE30Xa:

HV2:	Htroc_HV2_fw:	5`-ATT ACT TAC ACT GAT TGT ACA G-3`
	Htroc_HV2_rev:	5`-TTA AGC TTC ATT GTA AAT ATT CTT CTG G-3`
HV3-PAYD:	Htroc_HV3_fw1:	5`-ATT ACT TAC ACT GAT TGT ACA G-3`
	Htroc_HV3_rev1:	5`-TTA AGC TTA ATC ATA AGC GTC TTC TGG-3`
HV3-PAFN:	Htroc_HV3_fw2:	5`-ATT ACT TAC ACT GAT TGT ACA G-3`
	Htroc_HV3_rev2:	5`-TTA AGC TTA ATT AAA AGC GTC TTC TGG-3`
HLF1:	Htroc_HLF1V_fw:	5`-ATT GTT TAT GGA CCC TGC TCA GAA AAT-3`
	Htroc_HLF1V_rev:	5`-TTA AGC TTA TTT ATC ATC ATC GTC GTC-3`
HLF2:	Htroc_HLF2_fw:	5`-ATT GTT TTT AAA CCC TGC TCG GAA ACT-3`
	Htroc_HLF2_rev:	5`-TTA AGC TTA TTT ATT ATC GTC GTC GTC-3`
HLF-Hyb:	Htroc_HLF1V_fw:	5`-ATT GTT TAT GGA CCC TGC TCA GAA AAT-3`
	Htroc_HLF2_rev:	5`-TTA AGC TTA TTT ATT ATC GTC GTC GTC-3`
